# Telehealth utilization during the COVID-19 pandemic: comparing breast cancer survivors to non-cancer patients and implications for current practice

**DOI:** 10.1007/s10549-026-07940-6

**Published:** 2026-03-24

**Authors:** Lashez A. Hawkins, Ruvarashe P. Rumano, Sharnell S. Smith, Chloe M. Beverly-Hery, James L. Fisher, Akia Clark, Victoria Champion, Bridget A. Oppong, Electra D. Paskett

**Affiliations:** 1 Central State University, Wilberforce, OH USA; 2 The Ohio State University College of Public Health, Columbus, OH USA; 3 Department of Surgery, The Ohio State University Wexner Medical Center, Columbus, OH USA; 4 The Ohio State University James Comprehensive Cancer Center, Columbus, OH USA; 5 Indiana University Simon Comprehensive Cancer Center, Indianapolis, IN USA

**Keywords:** Breast cancer, COVID-19, Telehealth

## Abstract

**Background:**

During the 2019 coronavirus (COVID-19) pandemic, in-person medical visits changed due to social distancing guidelines. Breast cancer (BC) patients, needing ongoing treatment or surveillance, faced increased challenges in accessing care. Telehealth became essential for providing convenient and cost-effective care while minimizing COVID-19 transmission. BC survivors, however, often value in-person visits for clinical exams. This study aimed to compare telehealth participation between patients with a history of BC and women without cancer history.

**Methods:**

Adults aged 18 and older, including cancer patients, survivors, caregivers, and healthy volunteers, primarily from Ohio, were recruited with attention and inclusion of underserved and minority populations to complete a survey about COVID-19-related beliefs, practices, and knowledge. Recruitment involved (1) re-contacting participants from previous OSU studies (2) outreach via community partners and listservs to invite additional participants and caregivers. Sociodemographic characteristics by BC status were calculated using Chi square tests. Univariable and multivariable logistic regressions modeled association between the outcome of interest, telehealth participation, and BC status accounting for race, ethnicity, age, education, marital status, rurality, and insurance status.

**Results:**

The final sample included 2265 participants, with 43.7% having a history of BC. Significant demographic differences were observed between participants with and without a history of breast cancer. Those with a previous BC diagnosis were younger on average (55.6 vs. 57.9 years, *p <* .001), had higher levels of educational attainment (*p <* .001), were less often married and more often divorced/widowed/separated (*p =* .037), and were more likely to have private insurance only and less likely to have both public and private coverage (*p <* .001). Telehealth use was lower during COVID-19 among BC survivors (41.5%) vs. those without cancer (63.9%) (*p <* 0.001). In a multivariable model, factors that were statistically significantly associated with increased utilization of telehealth were Black race (OR = 1.90, p-value 0.036), having some college education (OR = 1.50, p-value 0.034), being married (OR = 1.61, p-value 0.009), and being currently employed (OR = 1.25, p-value 0.050). BC diagnosis or survivor status was associated with decreased odds of telehealth use (OR: 0.72, *p =* 0.023). Among breast cancer patients with complete data (n = 645 of the 989 total), more than half used telehealth, with video visits being slightly more common than phone visits. Logistic regression analyses revealed increased telehealth use among patients with a history of BC was associated with age > 70, while decreased participation in telehealth was associated with higher educational status and having undergone surgical treatment.

**Conclusion:**

We found that Black race, having some college education, being married, and being employed were significantly associated with increased telehealth participation during the COVID-19 pandemic. Interestingly, BC diagnosis was associated with reduced odds of telehealth use. Subgroup analyses of patients with a history of BC showed decreased use of telehealth to be associated with higher education and recent surgery for BC. Further investigation is needed to understand the acceptability and barriers to telehealth among BC survivors, as this modality continues to play an expanding role in oncology care delivery in the post-pandemic era.

**Supplementary Information:**

The online version contains supplementary material available at 10.1007/s10549-026-07940-6.

## Introduction

In the United States, breast cancer (BC) is the most commonly diagnosed cancer in women, with an estimated 310,720 new invasive cases and 42,250 deaths in 2024, corresponding to an incidence of about 131 and a mortality of 19 per 100,000 women [1]. In Ohio, approximately 10,071 new breast cancer cases and 1698 deaths occur annually [1]. The coronavirus (COVID-19) pandemic prompted a paradigm shift in healthcare delivery, aiming to maintain essential care while minimizing viral transmission. Since 2020, the United States has reported over 100 million COVID-19 cases and more than 1.1 million deaths, including roughly 3.7 million cases and 43,896 deaths in Ohio [1]. Early in the pandemic, declines were observed across the breast cancer care continuum—including screening, diagnosis, treatment, and surveillance—disrupting control efforts and potentially impacting breast cancer-related mortality [2]. To mitigate transmission risks, healthcare systems rapidly transitioned from in-person visits to widespread telehealth adoption—embraced by diverse patient populations across both oncology and non-cancer outpatient care. [3–5] Telehealth, delivered via phone or Internet-based platforms, encompasses a broad range of clinical and non-clinical services, including consultations, diagnosis, health education, remote monitoring, and administrative functions [6]. These services are enabled by information and communication technologies (ICT), such as video conferencing, mobile applications, and secure messaging [6]. Although telemedicine existed prior to the pandemic, its use and scholarly evaluation remained limited until COVID-19 accelerated both [7]. The expansion of telehealth aimed to enhance patient and provider safety, reduce travel and wait times, improve convenience, and support continuity of care, benefits that remain relevant in the post-pandemic landscape. Telehealth may also mitigate time toxicity, the cumulative burden of time spent on in-person visits, travel, and care coordination, which is increasingly recognized as a critical factor in patient-centered cancer care [8–10].

Concerns have been raised about telehealth acceptance among patients with a history of BC. Several studies found that many patients preferred in-person visits focused on BC treatment (including surgical management and preparation as well as broader treatment options such as radiation, chemotherapy, targeted therapy, and immunotherapy), valuing face-to-face interactions—particularly for important discussions and breast exams—which offered greater comfort and a stronger personal connection with their providers [3]. Numerous studies have explored the efficacy of telehealth and the satisfaction levels of patients with a history of BC amidst the COVID-19 pandemic [3, 4]. While active treatments such as surgery, chemotherapy, and radiation therapy required in-person visits, survivorship follow-ups and supportive care programs emerged as ideal candidates for virtual delivery. A retrospective study examined the utilization of BC survivorship services during the pandemic [11, 12]. The survivorship programs (SPs) were virtual and provided clinical and non-clinical programming such as physical rehabilitation, emotional and psychosocial support, nutrition, and exercise programming. The most significant shift in participation rates among non-clinical SPs during the pandemic was observed in the utilization of exercise content, which surged by 220% from 2019 to 2020 [11, 12]. Another descriptive study of breast cancer survivors (stage 0–III) found high satisfaction and strong information recall after nurse practitioner led telehealth SCP visits, indicating the potential of telehealth to support survivorship care delivery [13].

The current study aims to enhance comprehension regarding the sociodemographic and clinical factors related to utilization of telehealth services among female BC survivors, compared to those without a history of cancer, during the COVID-19 pandemic.

## Methods

### Overview

This study was part of an NCI-funded initiative conducted in conjunction with 16 other NCI-designated Cancer Centers, the IC-4 (Impact of COVID-19 on the Cancer Continuum Consortium) [14]. The initiative was funded to work collectively to develop core survey items and implement population surveys in the respective catchment areas. The overall goal of the IC-4 was to assess how differences in demographics (rural/urban residence, age, gender, race, educational attainment) impact engagement in cancer preventive behaviors (e.g., tobacco cessation, screening, diet) and cancer management/survivorship behaviors (e.g., adherence to treatment, adherence to surveillance, access to health services) in the context of COVID-19 environmental constraints (e.g., social distancing, employment, mental health). Each site had its own theoretical framework and survey methods. Our site used the IC-4 core set of common data elements, augmented with questions of interest to the local team, with remote data collection methods to include many unique and diverse populations. This study was approved by the OSU Institutional Review Board in June 2020.

#### Theoretical framework

This study was grounded in the Health Belief Model (HBM). According to the HBM, individuals’ change in health behaviors depends on a series of health beliefs, include individuals’: (1) perceived susceptibility to COVID-19 exposure, (2) perceived severity of the consequences of contracting COVID-19 (e.g., hospitalization or death), (3) perceived benefits of the effectiveness of the proposed COVID-19 prevention measures, (4) perceived barriers to executing the proposed prevention measures, (5) cue to the proposed prevention actions, and (6) self-efficacy in the person's ability successfully perform COVID-19 prevention measures [15]. 

#### Survey development

**Fig. 1:**
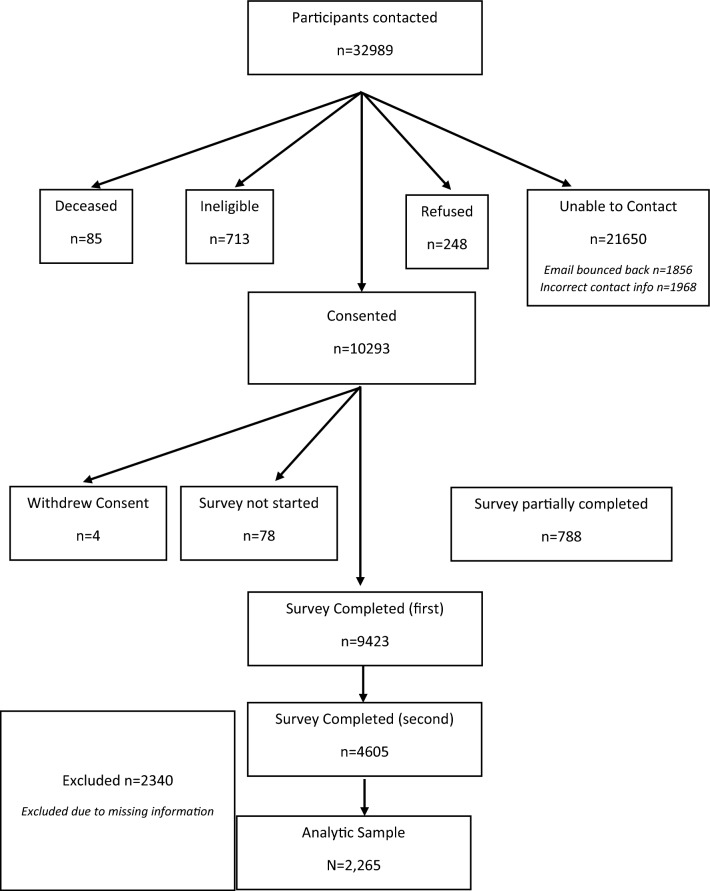
COVID-19 Survey recruitment flow chart

The survey elements (See Online Appendix 1) were finalized in conjunction with other members of the IC-4. The survey included individual behaviors related to mitigation of COVID-19 transmission, the challenges related to social distancing, self/family isolation, stress, and health behaviors that are highly relevant to cancer and other chronic diseases (i.e., type, duration and location of physical activity, tobacco/marijuana or alcohol use, vaping/e-cigarette use, exposure to second hand smoke, nutrition/diet, health information-seeking and participation in clinical trials, and access to health services). Questions also assessed perceived stigma related to COVID-19 with respect to different population groups and variables, such as health literacy and mental health, suspected of moderating these influences. Moreover, we assessed the impact of children being out of school and employment challenges (i.e., remote working and unemployment etc.), as well as the influence of social media on information, knowledge, behaviors, and attitudes (See Fig. 1).

### Sample selection

Participants who agreed to be re-contacted from previous studies were asked to participate in this study with a wide variety of populations, including patients with and without a history of cancer. Eligible participants were adults aged 18 years or older who consented to take part in the study. To ensure the inclusion of the most vulnerable, underserved, and minority populations, we sought to recruit healthy adult volunteers, cancer patients, cancer survivors, and cancer patients and caregivers, mainly from Ohio, with some from Indiana and other states. This was achieved by employing two recruitment strategies. First, we identified and contacted individuals who previously participated in studies conducted at OSU and consented to being contacted for future research projects. In addition, we invited cancer patients and survivors to nominate their primary caregivers to participate in the study. The list of previous research projects conducted at OSU included the Rural Interventions for Screening Effectiveness (RISE) study (R01 CA196243), the Community Initiative Towards Improving Equity and Health Status (CITIES) cohort (Supplement to P30CA016058), the Ohio State University Center of Excellence in Regulatory Tobacco Science (CERTS) cohort (P50CA180908), and members of the Total Cancer Care (TCC) cohort (P30CA016058). Second, to further enhance the representative of our study sample and ensure the inclusion of minority and underserved communities, we utilized our community partners and listservs to send tailored email invitations. Although our recruitment pool included adults with and without a history of cancer, the analysis focuses specifically on female BC survivors and a comparison group without a cancer history, consistent with the study aim.

### Interview/data collection

We utilized several data collection methods, including Web, phone, and mailed surveys. Respondents with valid emails received an initial survey invitation via email along with three reminders seven days apart. The first survey was completed from June to November 2020, then the second from March to July 2020. All participants were initially screened using an eligibility form to confirm their current Ohio or Indiana residence before conducting the survey. Participants were able to save the Web survey and resume it at a later time. Those who partially completed the Web survey received an email reminder one week after they last accessed the survey. A trained interviewer contacted participants without an email address and those with invalid emails on file by phone. Participants who were initially reached by phone were offered the option to complete the survey over the phone or online. We mailed a cover letter and a paper survey with a self-addressed, stamped return envelope to participants who requested a mailed survey. For non-English-speaking participants, a bilingual staff member administered the survey in the appropriate language (e.g., Spanish). Participants were offered a $10 gift card upon completion of the survey. All data were collected and managed using the Research Electronic Data Capture (REDCap) secure Web-based application hosted at OSU.

### Measures

To evaluate the impact of BC survivorship on health beliefs, behaviors, and telehealth utilization, we evaluated the following variables: age, race, education, income level, marital status, health insurance type, geographic residence (rural vs. urban), health and medical care, cancer history, BC history, and telehealth usage. Race was categorized as follows: “White,” “Black or African American,” “American Indian or Alaskan Native,” “Asian or Asian American,” “Native Hawaiian or Pacific Islander,” and “other.” Education level was listed as follows: “High School or Less,” “Some College/Associate,” “BS/BA,” “MS/MA or More,” and “Missing/Refused/Don’t Know,” based on original responses such as “Less than high school,” “Some high school, no diploma,” “GED,” “High school graduate,” “Some college but no degree,” various associate degrees, bachelor’s degrees, master’s degrees, professional degrees, and doctorates. Income-level amounts were “ < $35 K,” “$35 K–$49,999,” “$50 K–$74,999,” and “$75 K + .” Marital status was determined by the following selections: “Single, never been married,” “Married,” “Not married but living together,” “Separated,” “Divorced,” “Widowed,” or “Other.” Health insurance types were “Medicaid,” “Private health insurance,” “Medicare,” “Medicare plus a supplemental policy,” and “Military/ VA.” Responses were grouped into “no insurance,” “private,” “private and public,” and “public insurance.”

Health and medical care data were assessed by asking participants if they had postponed or skipped a healthcare visit, and status of various health related visits (for example, screening, chronic illness, new/changing health concern, urgent care, or other) due to COVID-19.

Data related to the pandemic was recorded using several questions. Participants were asked if a doctor had ever diagnosed them with cancer. Those who indicated “yes” to a history of cancer were asked additional questions about their active cancer-related treatment during COVID-19 (i.e., surgery and radiation status). Further information was gathered on their diagnosis from medical record review. BC-related variables that are included in this analysis consisted of the following: years since BC diagnosis, SEER stage, and treatment related variables (surgery and radiation).

Telehealth usage was measured by asking the participants if they had completed a telehealth visit since the pandemic. If they answered “yes,” participants were asked to specify if it was by phone or video. Additional variables related to telehealth usage were also included, such as access to Internet, confidence while using devices, and participation in telehealth before the COVID-19 pandemic.

### Data analysis

Demographic and clinical factors were summarized according to utilization of telehealth services using descriptive statistics, with frequencies and percentages for categorical variables (e.g., race) and means and standard errors for continuous variables (e.g., age). Differences between those who utilized telehealth services and those who did not were calculated using Chi square tests (Fisher’s exact test, when appropriate). Univariable logistic regressions were used to calculate odds ratios reflecting differences in odds of telehealth utilization among BC survivors (compared to those not previously diagnosed with any cancer) and for categories of potentially confounding factors. Multivariable logistic regression was used to develop a model identifying factors which were at least marginally significantly associated with telehealth utilization, after adjustment for confounding by additional factors in the model. These factors, identified from responses to the COVID-19 survey due to their potential impact on telehealth utilization, were included in the multivariable model (Table 1) to assess their independent associations with telehealth use. Factors with at least marginally statistically significant (*p <* 0.15) associations in univariate analyses were initially included in the multivariable model. Factors were removed individually, first with the weakest factor, until all factors remaining in the model were significantly associated with telehealth utilization or were important to the model in such a way as to substantively alter association(s) between additional factor(s) in the model and telehealth utilization by 15% or more. Therefore, the final model contained factors that were independently associated with telehealth utilization or important confounders of associations with additional model factors. A subgroup analysis of only those reporting a history of BC was conducted with univariable and multivariable logistic regression for factors associated with telehealth participation. All statistical analyses were conducted using IBM SPSS Statistics version 29.0.1.0 (IBM Corp, Armonk, NY). A two-sided p-value of less than 0.05 was considered statistically significant.

**Table 1:** Odds ratios and 95% confidence intervals from a multivariable logistic regression reflecting associations between odds of having ever participated in a telehealth visit since the COVID-19 pandemic and selected factors

	Odds ratio	95% CI	p-value
Race			
White (referent)	/	/	/
Black/African American	1.90	1.04–3.47	0.036
Asian	0.55	0.24–1.27	0.162
Other/multiple	0.68	0.36–1.31	0.251
Missing/refused/don’t know	1.21	0.28–5.30	0.803
Education			
High school or less (referent)	/	/	/
Some college/associate	1.50	1.03–2.17	0.034
BS/BA	1.25	0.86–1.80	0.247
MS/MA or more	1.31	0.90–1.90	0.166
Missing/refused/don’t know	7.50	0.17–333.3	0.299
Marital status			
Single/never married (referent)	/	/	/
Married/living as married	1.61	1.13–2.31	0.009
Div/Wid/Sep/Other	1.40	0.91–2.13	0.129
Missing/refused/don’t know	0.88	0.05–14.5	0.929
Breast cancer diagnosis			
No (referent)	/	/	/
Yes	0.72	0.55–0.96	0.023
Vehicle access			
No (referent)	/	/	/
Yes	0.94	0.02–0.41	.002
Missing/refused/don’t know	Not estimable		
Currently working?			
No (referent)	/	/	/
Yes	1.25	1.00–1.56	0.050
Missing/refused/don’t know	0.75	0.20–2.88	0.679
Postponed/skipped any healthcare in past year due to COVID			
No (referent)	/	/	/
Yes	2.34	1.84–2.99	< .001
Missing/refused/don’t know	0.17	0.01–2.84	0.219
Confidence level using computers, smartphones, devices			
Not at all (referent)	/	/	/
Only a little	0.18	0.05–0.74	0.017
Somewhat	0.29	0.06–1.09	0.067
Very	0.43	0.12–1.59	0.206
Missing/refused/don’t know	Not estimable		
Pre-COVID Telehealth Visit?			
No (referent)	/	/	/
By phone only	0.53	0.35–0.80	0.003
By video only	0.71	0.44–1.14	0.152
By phone and video	0.49	0.29–0.82	0.007
Missing/refused/don’t know	0.25	0.01–6.93	0.416

## Results

Demographic characteristics of our sample (N = 2265) according to BC diagnosis versus no diagnosis of cancer at any site are described in Table 2. Of the 2265 participants, 989 (43.7%) had a previous BC diagnosis and 1276 (56.3%) did not have a history of any cancer. Age, education, marital status, and insurance status differed significantly between the groups. Participants with BC were more likely to be among ages 50–59 years old compared to those without a cancer diagnosis (31.6% vs. 26.4%, *p <* 0.0001). Also, participants with BC were more likely to have a higher level of education compared to those without cancer (BS/BA: 32.0% vs. 27.1%, *p =* 0.001). Additionally, participants with BC compared to those with no cancer diagnosis were significantly more likely to be divorced/widowed/separated/other (19.3% vs. 15.6%, *p =* 0.037). Participants with BC were also more likely to have private insurance (67.2% vs. 56.8%, *p =* 0.001) compared to those with no cancer diagnosis.

**Table 2:** a Participant demographics according to breast cancer diagnosis. b Telehealth visit since pandemic according to breast cancer diagnosis

	Breast cancer diagnosis (N = 989, 43.7%)	No cancer diagnosis (N = 1276, 56.3%)	p-value
a			
Age mean (standard error)	55.58 (0.40)	57.90 (0.35)	< 0.001
Age group			
< 40	122 (12.3%)	127 (10.0%)	
40–49	142 (14.4%)	169 (13.2%)	
50–59	313 (31.6%)	337 (26.4%)	
60–69	300 (30.3%)	414 (32.4%)	
70 +	112 (11.3%)	229 (17.9%)	
Race			.262
White	890 (90.0%)	1151 (90.2%)	
Black/African American	42 (4.2%)	68 (5.3%)	
Asian	16 (1.6%)	22 (1.7%)	
Other/multiple	31 (3.1%)	29 (2.3%)	
Missing/refused/don’t know	10 (1.0%)	6 (0.5%)	
Hispanic Ethnicity			.065
No	957 (96.8%)	1251 (98.0%)	
Yes	22 (2.2%)	21 (1.6%)	
Missing/refused/don’t know	10 (1.0%)	4 (0.3%)	
Education			< .001
High school or less	78 (7.9%)	161 (12.6%)	
Some college/associate	279 (28.2%)	424 (33.2%)	
BS/BA	316 (32.0%)	346 (27.1%)	
MS/MA or more	309 (31.2%)	343 (26.9%)	
Missing/refused/don’t know	7 (0.7%)	2 (0.2%)	
Income group			.235
< $35 K	95 (9.6%)	131 (10.3%)	
$35 K-$49,999	72 (7.3%)	110 (8.6%)	
$50 K-$74,999	163 (16.5%)	199 (15.6%)	
$75 K +	536 (54.2%)	646 (50.6%)	
Missing/refused/don’t know	123 (12.4%)	190 (14.9%)	
Marital status			.037
Single/never married	94 (9.5%)	120 (9.4%)	
Married/living as married	700 (70.8%)	956 (74.9%)	
Div/Wid/Sep/Other	191 (19.3%)	199 (15.6%)	
Missing/refused/don’t know	4 (0.4%)	1 (0.1%)	
Insurance status			< .001
No insurance	24 (2.4%)	21 (1.6%)	
Public only	83 (8.4%)	130 (10.2%)	
Private only	665 (67.2%)	725 (56.8%)	
Public and private	208 (21.0%)	385 (30.2%)	
Missing/refused/don’t know	9 (0.9%)	15 (1.2%)	
Rural urban continuum code			.137
Non-metro	352 (35.6%)	501 (39.3%)	
Metro	635 (64.2%)	770 (60.3%)	
Missing	2 (0.2%)	5 (0.4%)	
b			
Telehealth visit since pandemic			< .001
No	579 (58.5%)	460 (36.1%)	
Yes	410 (41.5%)	816 (63.9%)	
By Phone*	229 (23.2%)	463 (36.3%)	
By Video*	299 (30.2%)	622 (48.7%)	

^*^Overlapping counts

BC status with regard to any telehealth participation during the COVID-19 pandemic is described in Table 2B. Of these participants with BC, 410 (41.5%) participated in a telehealth visit since the pandemic and 579 (58.5%) did not. Of those BC participants who did participate in telehealth, 229 (55.9%) participants accessed their telehealth visit by phone and 299 (72.9%) accessed by video. In addition, having a telehealth visit since the pandemic was lower among BC survivors compared to those without any cancer diagnosis (41.5% vs. 63.9%, *p <* 0.001).

Characteristics of our sample according to participation in telehealth visits since the COVID-19 pandemic are described in Table 3. Of these participants, 1226 (54.1%) participated in a telehealth visit since the pandemic and 1039 (45.9%) did not. There was a significant difference in age, with a mean of 55.90 years for those who participated in a telehealth visit, compared to mean of 57.72 years for those who did not (*p <* 0.001). The groups also differed between self-reported racial categories. Those who participated in telehealth compared to those who did not use telehealth were more likely to be Black/African American (5.8% vs. 3.8%, *p =* 0.02). Additionally, when analyzing the entire sample (Table 3), a higher percentage of those who used telehealth compared to no telehealth did not have a cancer diagnosis (66.6% vs. 44.3%, *p <* 0.001).

**Table 3:** Participant demographics according to having ever participated in a telehealth visit since the COVID-19 pandemic

	Participated in a telehealth visit since pandemic (N = 1226, 54.1%)	Not participated in a telehealth visit since pandemic (N = 1039, 45.9%)	p-value
Age mean (standard error)	55.90 (0.40)	57.72 (0.35)	< 0.001
Age group			.010
< 40	120 (9.8%)	129 (12.4%)	
40–49	152 (12.4%)	159 (15.3%)	
50–59	352 (28.7%)	298 (28.7%)	
60–69	395 (32.2%)	319 (30.7%)	
70 +	207 (16.9%)	134 (12.9%)	
Race			.020
White	1107 (90.3%)	934 (89.9%)	
Black/African American	71 (5.8%)	39 (3.8%)	
Asian	15 (1.2%)	23 (2.2%)	
Other/multiple	26 (2.1%)	34 (3.3%)	
Missing/refused/don’t know	7 (0.6%)	9 (0.9%)	
Hispanic ethnicity			.099
No	1203 (98.1%)	1005 (96.7%)	
Yes	18 (1.5%)	25 (2.4%)	
Missing/refused/don’t know	5 (0.4%)	9 (0.9%)	
Education			.315
High school or less	137 (11.2%)	102 (9.8%)	
Some college/associate	396 (32.3%)	307 (29.5%)	
BS/BA	345 (28.1%)	317 (30.5%)	
MS/MA or more	342 (27.9%)	310 (29.8%)	
Missing/refused/don’t know	6 (0.5%)	3 (0.3%)	
Income group			.142
< $35 K	139 (11.3%)	87 (8.4%)	
$35 K-$49,999	98 (8.0%)	84 (8.1%)	
$50 K-$74,999	185 (15.1%)	177 (17.0%)	
$75 K +	630 (51.4%)	552 (53.1%)	
Missing/refused/don’t know	174 (14.2%)	139 (13.4%)	
Marital status			.473
Single/never married	115 (9.4%)	99 (9.5%)	
Married/living as married	883 (72.0%)	773 (74.4%)	
Div/Wid/Sep/Other	225 (18.4%)	165 (15.9%)	
Missing/refused/don’t know	3 (0.2%)	2 (0.2%)	
Insurance status			.293
No insurance	23 (1.9%)	22 (2.1%)	
Public only	129 (10.5%)	84 (8.1%)	
Private only	734 (59.9%)	656 (63.1%)	
Public and private	327 (26.7%)	266 (25.6%)	
Missing/refused/don’t know	13 (1.1%)	11 (1.1%)	
Rural urban continuum code			.760
Non-Metro	467 (38.1%)	386 (37.2%)	
Metro	756 (61.7%)	649 (62.5%)	
Missing	3 (0.2%)	4 (0.4%)	
Breast cancer diagnosis			< .001
No	816 (66.6%)	460 (44.3%)	
Yes	410 (33.4%)	579 (55.7%)	

Results from univariable logistic regression analysis are presented in Table 4. Women with a BC diagnosis were less likely to use telehealth compared to those without a history of cancer (OR: 0.40, 95% CI: 0.34–0.47, *p <* 0.001). Women aged 70 and older had higher odds of a telehealth visit compared to those under 40 years (OR:1.67, 95% CI: 1.19–2.31, *p =* 0.003). Compared with white women, odds of a telehealth visit were significantly higher among Black women (OR: 1.54, 95% CI: 1.03–2.29, *p =* 0.036). There was no significant difference in the use of telehealth by ethnicity. Compared to individuals earning < $35,000, those earnings $50,000–74,999 (OR: 0.65, 95% CI: 0.47–0.92, *p =* 0.014) and $75,000 + (OR: 0.71, 95% CI: 0.53–0.96, *p =* 0.024) were less likely to use telehealth. Factors also associated with lower odds of using telehealth were having access to a vehicle (OR = 0.13, 95% CI: 0.03–0.54, *p =* 0.005), having postponed or skipped any healthcare in the past year due to COVID (OR = 0.59, 95% CI: 0.49–0.71, *p <* 0.001), having reliable telephone connection at home (OR = 0.51, 95% CI: 0.27–0.95, *p =* 0.033), and higher confidence level of using technology (OR = 0.31, 95% CI: 0.10–0.95, *p =* 0.041). Participation in a pre-COVID telehealth visit by video increased likelihood of using telehealth (OR: 1.64, 95% CI 1.07–2.54, *p =* 0.024). We did not observe statistically significant differences in odds of a telehealth visit by educational attainment, marital status, insurance, and residence in a metropolitan county.

**Table 4:** Odds ratios and 95% confidence intervals from univariable logistic regressions reflecting associations between odds of having ever participated in a telehealth visit since the COVID-19 pandemic and selected factors

	Odds ratio	95% CI	p-value
Breast cancer diagnosis			
No (referent)	/	/	/
Yes	0.40	0.34–0.47	< .001
Age (per year increase)			
Age group			
< 40 (referent)	/	/	/
40–49	1.03	0.74–1.43	0.873
50–59	1.28	0.95–1.70	0.110
60–69	1.33	1.000–1.78	0.052
70 +	1.67	1.19–2.31	0.003
Race			
White (referent)	/	/	/
Black/African American	1.54	1.03–2.29	0.036
Asian	0.55	0.30–1.06	0.074
Other/multiple	0.65	0.40–0.1.08	0.097
Missing/refused/don’t know	0.66	0.24–1.80	0.405
Hispanic ethnicity			
No (referent)	/	/	/
Yes	0.60	0.33–1.11	0.103
Missing/refused/don’t know	0.46	0.16–1.40	0.170
Education			
High school or less (referent)	/	/	/
Some college/associate	0.96	0.71–1.29	0.790
BS/BA	0.81	0.60–1.09	0.170
MS/MA or More	0.82	0.61–1.11	0.200
Missing/refused/don’t know	1.50	0.36–6.10	0.580
Income group			
< $35 K (referent)	/	/	/
$35 K-$49,999	0.73	0.49–1.09	0.120
$50 K-$74,999	0.65	0.47–0.92	0.014
$75 K +	0.71	0.53–0.96	0.024
Missing/refused/don’t know	0.78	0.55–1.11	0.170
Marital status			
Single/never married (referent)	/	/	/
Married/living as married	0.98	0.74–1.31	0.908
Div/Wid/Sep/Other	1.17	0.84–1.64	0.349
Missing/refused/don’t know	1.29	0.21–7.89	0.782
Insurance status			
No insurance (referent)	/	/	/
Public only	1.47	0.77–2.80	0.243
Private only	1.07	0.59–1.94	0.823
Public and private	1.18	0.64–2.16	0.601
Missing/refused/don’t know	1.13	0.42–3.05	0.809
Rural urban continuum code			
Non-metro (referent)	/	/	/
Metro	0.96	0.81–1.14	0.664
Missing	0.62	0.14–2.79	0.533
Vehicle access			
No (referent)	/	/	/
Yes	0.13	0.03–0.54	.005
Missing/refused/don’t know	.001	.000-.006	< .001
Days typically left home for work in past 3 weeks			
No	/	/	/
Yes	0.92	0.77–1.10	0.351
Missing/refused/don’t know	0.72	0.22–2.36	0.584
Postponed/skipped any healthcare in past year due to COVID			
No (referent)	/	/	/
Yes	0.59	0.49-.0.71	< .00.1
Missing/refused/don’t know	1.19	0.93–1.53	0.174
Internet access at home			
No (referent)	/	/	/
Yes	0.77	0.39–1.54	0.461
Missing/refused/don’t know	1.19	0.58–2.43	0.640
Reliable telephone connection at home			
No (referent)	/	/	/
Yes	0.51	0.27–0.95	0.033
Missing/refused/don’t know	0.81	0.42–1.55	0.524
Confidence level using computers, smartphones, devices			
Not at All (referent)	/	/	/
Only a little	0.41	0.12–1.36	0.144
Somewhat	0.56	0.78–1.74	0.313
Very	0.31	0.99–0.95	0.041
Missing/refused/don’t know	0.58	0.18–1.82	0.347
Pre-COVID telehealth visit?			
No (referent)	/	/	/
By phone only	1.30	0.90–1.89	0.168
By video only	1.64	1.07–2.54	0.024
By phone and video	1.25	0.78–2.02	0.353
Missing/refused/don’t know	1.61	1.27–2.04	< .001

The factors of interest included race, age, marital status, breast cancer diagnosis, current employment status, whether the participant postponed or skipped any healthcare in the past year due to COVID-19, confidence in using computers, smartphones, and other devices, and whether the participant had a telehealth visit prior to the COVID-19 pandemic. History of BC was associated with reduced odds of telehealth use (OR: 0.72, 95% CI: 0.55–0.96, *p =* 0.023). Other factors that were significantly associated with increased utilization of telehealth were Black race (OR: 1.90, 95% CI: 1.04–3.47, *p =* 0.036), having some college education (OR: 1.50, 95% CI:1.03–2.17, *p =* 0.034), being married (OR: 1.61, 95% CI: 1.13–2.31, *p =* 0.009), and being currently employed (OR: 1.25, 95% CI:1.00–1.56, *p =* 0.050). Also, those who postponed or skipped any healthcare visits due to the pandemic had an increased odds of participating in telehealth (OR: 2.34, 95% CI: 1.84–2.99, *p <* 0.001). Other factors that lowered odds of having a telehealth visit include having a vehicle, having “only a little” confidence with technology, or ever using telehealth prior to the COVID-19 pandemic.

Of the 989 patients with a history of BC, 645 patients had complete data on clinical factors (including SEER stage, surgery status, and radiation status) for the subgroup analysis. More than half of these patients used telehealth during the pandemic, with video visits slightly more common than phone visits. Specifically, 410 patients (64%) participated in at least one telehealth visit, and 73% of those used video. Age greater than 70 years (OR: 2.39, 95% CI: 1.13–5.08) increased telehealth participation. Higher educational attainment (OR: 0.37, 95% CI: 0.19–0.71) and surgical intervention (OR: 0.26, 95% CI: 0.07–0.91) were associated with decreased telehealth participation (Table 5).

**Table 5:** Frequencies, odds ratios, and 95% confidence intervals from univariable and multivariable logistic regressions reflecting associations between odds of having ever participated in a telehealth visit since the COVID-19 pandemic and selected factors among 645 female breast cancer participants of the ‘Impact of COVID-19’ Study

	Frequency*	Univariable	Multivariable
		OR (95% CI)	OR (95% CI)
Age group			
< 40 (referent)	71	/	/
40–49	99	0.78 (0.42–1.45)	0.72 (0.38–1.32)
50–59	194	1.01 (0.58–1.76)	0.97 (0.54–1.76)
60–69	204	1.25 (0.71–2.18)	1.29 (0.72–2.33)
70 +	77	2.48 (1.20–5.14)	2.39 (1.13–5.08)
Race			
White (referent)	584	/	
Black/African American	30	1.38 (0.62–3.07)	
Asian and other/multiple	24	1.18 (0.50–2.81)	
Hispanic ethnicity			
Non-hispanic (referent)	629		
Hispanic	10	0.57 (0.16–1.99)	
Education			
High school or less (referent)	57	/	/
Some college/associate	186	1.12 (0.58–2.18)	1.07 (0.55–2.11)
BS/BA	202	0.63 (0.33–1.21)	0.61 (0.32–1.19)
MS/MA or more	196	0.42 (0.22–0.81)	0.37 (0.19–0.71)
Income group			
< $35 K (referent)	71	/	
$35 K-$49,999	54	1.04 (0.49–2.21)	
$50 K-$74,999	99	0.52 (0.24–1.09)	
$75 K +	344	0.53 (0.27–1.02)	
Marital status			
Single/never married (referent)	63	/	
Married/living as married	441	1.43 (0.84–2.43)	
Divorced/widowed/separated/other	138	2.02 (1.09–3.73)	
Insurance status			
No Insurance (referent)	14	/	
Public only	59	0.84 (0.23–3.03)	
Private only	428	0.60 (0.18–1.93)	
Public and private	137	1.08 (0.32–3.66)	
Rural urban continuum code			
Non-metro (referent)	238	/	/
Metro	407	1.10 (0.80–1.53)	1.43 (0.98–2.09)
Currently working			
No (referent)	235	/	
Yes	268	0.84 (0.58–1.21)	
SEER summary stage at diagnosis			
In situ (referent)	61	/	
Localized	249	1.21 (0.68–2.49)	
Regional	131	1.12 (0.60–2.10)	
Distant	12	3.24 (0.65–16.11)	
Had surgery?			
No (referent)	21	/	/
Yes	620	0.28 (0.08–0.96)	0.26 (0.07–0.91)
Had radiation?			
No (referent)	245	/	
Yes	395	1.13 (0.82–1.58)	

^*^ Frequencies may not total to 645 due to missing values

## Discussion

The aim of this study was to understand the factors associated with telehealth usage among patients with a history of BC. We compared the usage of telehealth among BC survivors compared to those without cancer during the COVID-19 pandemic. We found that Black race, having some college education, being married, and being currently employed were characteristics significantly associated with increased participation. We subsequently evaluated the differences in telehealth participation among patients with a history of BC showing no differences in telehealth usage based on race, ethnicity, or income. Additionally, an important unique finding was the increased telehealth adoption among individuals over age 70, a demographic typically among the least likely to use the Internet. This trend may reflect the heightened awareness of COVID-19 risks for older adults, who were widely recognized as being at highest risk for severe outcomes, and the widespread public messaging encouraging them to limit in-person visits. Our findings are notable in terms of race, as many studies have demonstrated a lack of telehealth usage from vulnerable patient populations such as African Americans and Hispanics [16–19]. Similarly, a 2023 study reported factors that impact telehealth usage included age, gender, race, education, employment status, and income [17]. Their findings showed that while low-income, racial, and ethnic minority communities were at greater risk for health inequities, Hispanic communities were more likely to use telehealth than other ethnic groups in this study [17]. While our study primarily focused on Black/African American participants, we observed that Hispanic and Asian participants also demonstrated higher telehealth utilization compared to non-Hispanic White participants.

Our data also indicated that patients with a history of BC that had some college education were more likely to have utilized telehealth services. A supporting study with a subgroup analysis revealed that education levels were significantly associated with knowledge and prior experience with telehealth [20]. In our study, marital status was identified as a factor in telehealth usage among BC survivors, as well. This is consistent with previous research that has examined the benefits of marriage and healthcare [21, 22]. Marital status often serves as an indirect indicator of social support. A spouse can play a crucial role in encouraging patients to attend appointments and adhere to their care plans, including supporting the use of telehealth by helping set up technology, reminding patients of virtual visits, or providing assistance during the appointment itself [22]. Lastly, we found that being employed was associated with increased participation. Similarly, another study compared telehealth utilization between employed and non-employed patients, revealing that full-time employed patients were 15% more likely to use telehealth compared to unemployed patients [19]. 

Although there was not a statistically significant difference in telehealth usage based on home Internet access in this study, other research has shown that lack of reliable Internet is an important socioeconomic barrier to telehealth use. [23, 24] For instance, approximately 21 to 42 million Americans lack high-speed Internet access, which impacts their ability to utilize telehealth services. [24] These issues play a crucial role in delivering adequate telehealth care and decrease satisfaction among medically underserved communities [23–25]. In addition, low digital literacy creates significant barriers for many groups in accessing remote healthcare. While telehealth offers advantages, several studies have highlighted that many BC survivors were resistant to the idea and favored in-person visits. They valued face-to-face interactions, especially for crucial discussions and breast examinations, as these visits provided a sense of comfort and a stronger personal connection with their healthcare providers. [13, 26–28] The physical presence during appointments was perceived as more reassuring and conducive to building trust and understanding between patients and doctors, which they found lacking in virtual consultations. ^13^ While our study did not examine this directly, it is possible that patients without a prior trusting relationship with their provider may feel this effect more strongly, which they sometimes found lacking in virtual consultations. In our subgroup analysis of patients with a history of BC, surgical treatment was associated with reduced telehealth utilization. This may reflect that BC survivors experienced health issues requiring in-person visits, either due to new symptoms or ongoing aspects of their treatment, reducing the need or feasibility of virtual visits. However, because the survey did not capture whether participants were undergoing active treatment, it is possible that some BC survivors required in-person visits due to ongoing treatment or emerging health issues, which may partly explain their lower telehealth use. This phase of care often necessitates in-person encounters—for procedures such as drain removal—or reflects patient preferences for discussing results face-to-face.

### Strengths

This study had several strengths including a large sample size that allowed for the capture of data from a broad patient population. Since this study was part of the NCI-funded IC-4 initiative, it benefited from a collaborative approach with 16 other NCI-designated Cancer Centers in developing a comprehensive survey instrument. The study utilized multiple recruitment strategies to include underserved, vulnerable, and minority populations. The use of several remote data collection methods allowed for the inclusion of more patients. We had an option for non-English speaking participants to complete the survey in their preferred language. We also included primary caregivers, recognizing that their involvement in care coordination and technological support provides insight into factors influence telehealth uptake that may not be fully captured through patient responses alone. By capturing unique, timely, and relevant data, the study provided insights into BC patient preferences during the COVID-19 pandemic. These findings can lead to a comprehensive understanding of how the pandemic influenced healthcare choices and behaviors.

### Limitations

Several limitations were identified in our study. Firstly, the lack of diversity within our sample, primarily comprising White respondents, restricts the generalizability of our findings to more diverse demographic groups. Secondly, reliance on survey data introduces the potential for response bias, where participants may have responded in ways influenced by societal expectations or the context of the COVID-19 pandemic. Specifically, the preference for telehealth appointments during COVID-19, driven by the need for social distancing, may have skewed our results toward an overrepresentation of positive attitudes toward telehealth. Third, due to the survey-based nature of this study, we were unable to collect detailed clinical information regarding cancer treatment type or phase of care. Importantly, we did not distinguish between survivors receiving active systemic chemotherapy and those on long-term endocrine (hormone) therapy. This distinction is clinically meaningful, as patients undergoing chemotherapy often require more frequent in-person monitoring, management of treatment-related toxicities, and hospital-based care, whereas patients on oral endocrine therapy may be managed longitudinally in the outpatient setting over several years. Differences in treatment modality may therefore influence both the appropriateness of telehealth use and patient preferences. The absence of treatment-specific data limits our ability to contextualize telehealth utilization patterns and may partially explain the lower observed telehealth use among breast cancer survivors. Furthermore, we were not able to collect information on the reasons for telehealth visits, for example whether BC survivors were undergoing active treatment, which may have influenced their ability or need to use telehealth. This limitation may partly explain the lower observed telehealth use among BC survivors, as some aspects of BC care—particularly physical examination—cannot be adequately performed virtually. The inherent limitations of telehealth in replacing in-person clinical assessment should be considered when interpreting patient preferences (e.g., adherence improvement).

Fourth, because the survey was interviewer-administered for a small subset of the sample by a bilingual staff member, some social desirability bias may have been introduced, although the use of bilingual staff was necessary to ensure equitable participation among individuals with limited English proficiency. Lastly, the composition of our sample may not fully reflect real-world BC populations. Our recruitment strategy—rely on Ohio-based individuals previously enrolled in OSU research cohorts, community listservs, and caregiver nominations—may have introduced selection bias, as these participants may differ from the broader BC population in engagement, access to care, and technology comfort. Additionally, caregivers were included in the sample, and although their perspectives provide valuable insight, their inclusion may influence the overall findings and should be interpreted with this context in mind. These factors collectively highlight the need for caution in generalizing our findings to broader populations or to clinical decision-making regarding telehealth use in BC care. Despite these limitations, understanding how the pandemic affected patient preferences in our catchment area remains important for informing future models of care.

## Conclusion

Based on our findings, further analysis focusing on the subgroup of women with a BC diagnosis would strengthen our understanding of factors that influence utilization of telehealth. This analysis could provide valuable insights into potential barriers to telehealth adoption, particularly in scenarios where patient considerations such as immunocompromised state (from chemotherapy) or societal factors such as lockdowns during the pandemic which may restrict in-person healthcare visits. Understanding these barriers is crucial for ensuring continued engagement in healthcare among BC survivors and identifying populations that may benefit from telehealth, while recognizing that individuals undergoing active treatment may still require frequent in-person visits. By exploring these dynamics, future studies can contribute to improving healthcare delivery and enhancing access to essential services for this vulnerable population.

## Supplementary Information

Below is the link to the electronic supplementary material.

Supplementary file1 (DOCX 18 KB)

## Data Availability

The data that support the findings of this study are available from the corresponding author upon request.

## References

[1] American Cancer Society. Breast Cancer Statistics | How Common Is Breast Cancer? www.cancer.org. Published January 17, 2024. https://www.cancer.org/cancer/types/breast-cancer/about/how-common-is-breast-cancer.html#:~:text=At%20this%20time%20there%20are

[2] Alagoz O, Lowry KP, Kurian AW et al (2021) Impact of the COVID-19 pandemic on breast cancer mortality in the US: estimates from collaborative simulation modeling. J Natl Cancer Inst 113(11):1484–1494. 10.1093/jnci/djab097 34258611 10.1093/jnci/djab097 PMC8344930

[3] Tang A, Neeman E, Kuehner GE et al (2022) Telehealth for preoperative evaluation of patients with breast cancer during the COVID-19 pandemic. Perm J 26(2):54–63. 10.7812/tpp/21.126 35933666 10.7812/TPP/21.126 PMC9662263

[4] García-Vigara A, Martín-González V, Carbonell JA et al (2023) The COVID-19 pandemic and the usability of telehealth in a midlife women’s health integrated care program. Maturitas 168(4):7–12. 10.1016/j.maturitas.2022.10.011 36370490 10.1016/j.maturitas.2022.10.011 PMC9633107

[5] Mclaughlin EJ, Ellett LC, Readman E, Mooney S (2022) Telehealth for gynaecology outpatients during the COVID‐19 pandemic: patient and clinician experiences. Aust N Z J Obstet Gynaecol. 10.1111/ajo.13510 35322405 10.1111/ajo.13510 PMC9111195

[6] Ryu S (2012) Telemedicine: opportunities and developments in member states: report on the second global survey on eHealth 2009 (Global Observatory for eHealth Series, Volume 2). Healthc Inform Res 18(2):153. 10.4258/hir.2012.18.2.153

[7] Shaver J (2022) The state of telehealth before and after the COVID-19 pandemic. Prim Care 49(4):517–530. 10.1016/j.pop.2022.04.002 36357058 10.1016/j.pop.2022.04.002 PMC9035352

[8] Oppong BA, Lustberg MB, Nolan TS et al (2022) Utilization of cancer survivorship services during the COVID-19 pandemic in a tertiary referral center. J Cancer Surviv 17(6):1708–1714. 10.1007/s11764-022-01231-x 35895236 10.1007/s11764-022-01231-x PMC9326963

[9] Teicher S, Whitney R, Liu R (2022) Breast cancer survivors’ satisfaction and information recall of telehealth survivorship care plan appointments during the COVID-19 pandemic. Oncol Nurs Forum 49(3):223–231. 10.1188/22.onf.223-231 35446836 10.1188/22.ONF.223-231

[10] Marcondes FO, Normand SLT, Le Cook B, Huskamp HA, Rodriguez JA, Barnett ML, Uscher-Pines L, Busch AB, Mehrotra A (2024) Racial and ethnic differences in telemedicine use. In JAMA Health Forum. 5(3):e240131–e240131. 10.1001/jamahealthforum.2024.0131 10.1001/jamahealthforum.2024.0131 PMC10960201 38517424

[11] Populations During the COVID-19 Pandemic: retrospective observational study. Journal of Medical Internet Research. 2023;25(25):e43604. 10.2196/436012 10.2196/43604 PMC10185335 37171848

[12] Reported Access to Telehealth: An Important and Unmeasured Social Determinant of Health. JCO Oncology Practice. 2023;19(12). 10.1200/op.23.00006 10.1200/OP.23.00006 37844269

[13] Jewett PI, Vogel RI, Ghebre R et al (2021) Telehealth in cancer care during COVID-19: disparities by age, race/ethnicity, and residential status. J Cancer Surviv. 10.1007/s11764-021-01133-4 34800257 10.1007/s11764-021-01133-4 PMC8605776

[14] Hofstede J, de Bie J, van Wijngaarden B, Heijmans M (2014) Knowledge, use and attitude toward eHealth among patients with chronic lung diseases. Int J Med Inform 83(12):967–974. 10.1016/j.ijmedinf.2014.08.011 25269992 10.1016/j.ijmedinf.2014.08.011

[15] Iwashyna TJ, Christakis NA (2003) Marriage, widowhood, and health-care use. Soc Sci Med 57(11):2137–2147. 10.1016/s0277-9536(02)00546-4 14512244 10.1016/s0277-9536(02)00546-4

[16] Kemp MT, Liesman DR, Brown CS et al (2020) Factors associated with increased risk of patient no-show in telehealth and traditional surgery clinics. J Am Coll Surg 231(6):695–702. 10.1016/j.jamcollsurg.2020.08.760 32891797 10.1016/j.jamcollsurg.2020.08.760 PMC7470818

[17] Clare CA (2021) Telehealth and the digital divide as a social determinant of health during the COVID-19 pandemic. Netw Model Anal Health Inform Bioinform. 10.1007/s13721-021-00300-y 33842187 10.1007/s13721-021-00300-y PMC8019343

[18] Sharma P, Patten CA (2022) A need for digitally inclusive health care service in the United States: recommendations for clinicians and health care systems. Perm J 26(3):149–153. 10.7812/tpp/21.156 35939597 10.7812/TPP/21.156 PMC9683741

[19] Office USGA. Telehealth in the Pandemic—How Has It Changed Health Care Delivery in Medicaid and Medicare? www.gao.gov. Published September 29, 2022. https://www.gao.gov/blog/telehealth-pandemic-how-has-it-changed-health-care-delivery-medicaid-and-medicare#:~:text=To%20prevent%20the%20spread%20of

[20] Cadili L, DeGirolamo K, Ma CSY et al (2021) The breast cancer patient experience of telemedicine during COVID-19. Ann Surg Oncol 29:2244–2252. 10.1245/s10434-021-11103-w 34820744 10.1245/s10434-021-11103-w PMC8612720

[21] Calip GS, Cohen A, Rohrer R et al (2023) Telemedicine use among patients with metastatic breast cancer during the COVID-19 pandemic: Differences by race, age, and region. Pharmacoepidemiol Drug Saf 32(1):66–72. 10.1002/pds.5541 36111444 10.1002/pds.5541 PMC10091805

[22] Kumar D, Gordon NP, Neeman E et al (2022) Patient preferences for telehealth versus in-person oncology visits. J Clin Oncol 40(28_suppl):386–386. 10.1200/jco.2022.40.28_suppl.386

[23] Ren L, Chen M, Jiang H, Wang Y, Xia L, Dong C (2024) Perceptions of adult patients with cancer towards telemedicine: A qualitative meta-synthesis. Asia-Pac J Oncol Nurs 11(2):100360. 10.1016/j.apjon.2023.100360 38293602 10.1016/j.apjon.2023.100360 PMC10825605

[24] Board I of M (US), NRC (US) NCP, Hewitt M, Herdman R, Holland J (2004) Psychosocial needs of women with breast cancer. National Academies Press, US 25009861

[25] Alyafei A, Easton-Carr R. The health belief model of behavior change. [Updated 2024 May 19]. In: StatPearls [Internet]. Treasure Island (FL): StatPearls Publishing; 2025 Jan-. Available from: https://www.ncbi.nlm.nih.gov/books/NBK606120/26.

[26] Atre SY, Soulos PR, Kuderer NM, Gross CP, Baum LVM, Dinan MA, Lustberg MB (2024) Characterization of time toxicity in older patients with metastatic breast cancer. Breast Cancer Res Treat 207(3):541–550. 10.1007/s10549-024-07379-727 38816556 10.1007/s10549-024-07379-7

[27] Gupta A, Brundage MD, Galica J, Karim S, Koven R, Ng TL, O’Donnell J, tenHove J, Robinson A, Booth CM (2024) Patients’ considerations of time toxicity when assessing cancer treatments with marginal benefit. Oncologist 29(11):978–985. 10.1093/oncolo/oyae18728 39045654 10.1093/oncolo/oyae187 PMC11546709

[28] Gupta A, Booth CM (2022) The time toxicity of cancer treatment. J Clin Oncol 40(15):1611–1615. 10.1200/JCO.21.02810 35235366 10.1200/JCO.21.02810

