# Supplementary material for: Telehealth utilization during the COVID-19 pandemic: comparing breast cancer survivors to non-cancer patients and implications for current practice
Source: Breast Cancer Res Treat. 2026 Mar 24;216(3):38. doi: 10.1007/s10549-026-07940-6 (PMC13013313; doi:10.1007/s10549-026-07940-6)
Supplement: Supplementary file 1 — Supplementary file1 (DOCX 18 KB) [file 10549_2026_7940_MOESM1_ESM.docx]

**Appendix 1. Survey elements by core constructs**

| **Survey elements by constructs** |
| --- |
| **Demographics**   - Neighborhood level: zip code, rural/urban - Individual level: age, gender, race/ethnicity educational attainment, income, occupation, employment status |
| **Perceived Susceptibility**   - Perceived susceptibility to COVID-19 |
| **Perceived Severity**   - Health consequences of COVID-19 - Perceived stigma related to COVID-19 diagnosis - Perceived financial impacts of COVID-19 |
| **Perceived Barriers/Benefits**   - Perceived stigma related to COVID-19 - Stress (including financial stressors), distress, discrimination/racism - Access to health information - Challenges related to social distancing (ability to get groceries, shortage of needed items, children home from school, ability to work from home, reduction of hours/pay, loss of employment, loss of health insurance) - Challenges related to the inability to engage in social distancing - Access to needed health services (not necessarily cancer related) – ability to get medications/get acute care, access & use of telehealth/phone visits - Ability to obtain cancer care (clinic visits, get recommended treatments, get recommended surveillance tests/procedures, modifications to regimens, participation/access to clinical trials, pain control/management) |
| **Cancer Prevention and Control Behaviors**   - Tobacco use (including vaping, e-cig)/Environmental tobacco exposure - Physical activity/inactivity - Fruit/vegetable intake - Alcohol intake - Breast, cervical, colorectal screening - Adherence to treatment/surveillance |
